# Increased risk of depression in patients with acquired sensory hearing loss: A 12-year follow-up study: Erratum

**DOI:** 10.1097/MD.0000000000006274

**Published:** 2017-02-24

In the article “Increased risk of depression in patients with acquired sensory hearing loss: A 12-year follow-up study”,^[1]^ which appeared in Volume 95, Issue 44 of *Medicine*, affiliations were listed incorrectly. Wei-Ting Hsu is affiliated with the Department of Otorhinolaryngology and Head and Neck Surgery, Mackay Memorial Hospital, Taipei, and Chih-Chao Hsu is affiliated with the Department of Psychiatry, Kaohsiung Veterans General Hospital, Kaohsiung.
